# Comparing the Efficiency of Three Protocols in Isolation of Cell Free Fetal DNA From Maternal Blood 

**Published:** 2017-09

**Authors:** Fatemeh Karami, Mohammad Reza Noori Daloii, Seddigheh Hantooshzadeh, Mohammad Hossein Modarressi

**Affiliations:** 1 Departement of Medical Genetics, Applied Biophotonics Research Center, Science and Research Branch, Islamic Azad University, Tehran, Iran; 2 Department of Medical Genetics, School of Medicine, Tehran University of Medical Sciences, Tehran, Iran; 3 Vali-e-Asr Reproductive Health Research Center, School of Medicine, Tehran University of Medical Sciences, Iran; 4 Departement of Medical Genetics, Science and Research Branch, Islamic Azad University, Tehran, Iran; Department of Medical Genetics, School of Medicine, Tehran University of Medical Sciences, Tehran, Iran

**Keywords:** DNA, Isolation, NIPD, Maternal Blood, Protocol

## Abstract

**Objective:** Recent advances in non-invasive prenatal diagnosis (NIPD) through cell free fetal DNA (cffDNA) has highlighted cffDNA purification as a critical initial step. Herein, we aimed to compare the efficiency of one proposed protocol with two commercial kits for isolation of cffDNA.

**Materials and methods:** cffDNA was isolated from whole blood of 50 normal pregnancies using one proposed manual protocol compared with QIAamp DNA Blood Mini and Bioneer Kits. Methylated DNA immunoprecipitation real time polymerase chain reaction (MeDIP-Real time PCR) was performed to quantify three fetal specific sequences.

**Results:** Maximum cffDNA quantity was obtained by suggested protocol (248.79 ± 14.07 ng/µl) and the best quality was achieved by Bioneer Kit (OD ratio: 260/280 nm/nm: 1.69 ± 0.09, 260/230 nm/nm: 1.15 ± 0.13) (p < 0.001). Enrichment of fetal specific sequences was significantly higher when proposed protocol was used to isolate cffDNA (p = 0.01).

**Conclusion:** Inhibitory effect of NaI on nucleases and double digestion of DNA associated proteins may be the main reasons behind the superiority of suggested protocol. Significantly higher amplification of fetal specific sequences in suggested protocol would be a strong evidence on recovery of small fetal fragments as demonstrated with its maximum total DNA quantity and amplification in different PCR reactions.

## Introduction

Introduction of various non-invasive prenatal diagnosis (NIPD) tests heralds for promises against conventional prenatal diagnosis associated with minimal, but finite risk of various fetal and maternal complications. NIPD have been extended to be used in determining fetal RhD statue (1) as well as fetal sex that is especially concerned in X-linked genetic disorders (2). Developing an appropriate and reproducible method for various steps of NIPD makes using them more available and robust in the early and safe detection of different prenatal morbidities.

Maternal blood is the main non-invasive source of fetal cells and cell free fetal DNAs (cffDNA) for different NIPD tests. Disruption of placental barriers during apoptosis process opens the door for fetal cells and DNAs to gain entry into the maternal circulation. It was shown that the quantity of fetal cells or cffDNA is a valuable and early biomarker of fetal abnormalities and second or third trimester placental complications such as pre-eclampsia (3). 

Fetal cells have longer half-life and remain in maternal circulation even until the next pregnancy. In contrast, cffDNA disappears from the maternal body within 2 hours postpartum, renders them a more appropriate and sensitive choice for non-invasive genetic diagnosis (4). However, scarcity of cffDNA in maternal blood (10-15% of all free DNA) hampered its isolation to be used in downstream steps of every NIPD described till now (5). Although, several methods and various commercial DNA isolation kits have been introduced for extraction of serum free DNA, obtained DNA doesn’t usually have enough quality and quantity. Primary strategies were relying on using of conservative substances such as formaldehyde to stabilize maternal white blood cells (WBCs) making them resistant against lysis. Initial high speed and double centrifugation of serum is another strategy which have been described to remove maternal WBCs from DNA isolation procedure (6, 7). However, the isolated cffDNA has still small quantity and quality that may make challenges in downstream steps of diagnosis and obtained results (8). 

Herein, we compared one proposed manual and two kit based methods of DNA isolation with each other to define which method is preferred for performing NIPD. To achieve this aim, cffDNA was isolated from whole blood of pregnant women with all of three protocols.

## Materials and methods

***Sample preparation: ***EDTA containing falcon tubes was used to collect 5 ml peripheral whole blood from 50 pregnant women whom were defined as high risk by doing first and second trimester screening tests and were referred for doing either chorionic villous sampling (CVS) or amniocentesis. Enrolled women were among 10-16 weeks of pregnancy (aged 18-40) and filled the informed consent form according to the code of Tehran University of Medical Sciences ethical committee (92-02-30-21577) for experiments involving human uniform requirements for manuscripts. All the included samples had a confirmatory karyotype analysis report. Twelve samples which have been drawn after amniocentesis and CVS or after 16 weeks of pregnancy were excluded from further assessments. 

All the whole blood samples were stored in -80˚C within 2 hours after venue puncture till the DNA isolation process being initiated.

The manual method of DNA isolation in comparison with QIAamp DNA Blood Mini Kit (Qiagen) and AccuPrepTM Genomic DNA Extraction Kit (Bioneer) were followed to extract total DNA from 1 milliliter of maternal whole blood. The quality and quantity of DNA was determined using NanoDrop ND-1000 spectrophotometer (NanoDrop Technologies, Wilmington, DE). Five micro liters of achieving DNA from all methods was amplified through standard PCR to confirm its quality again. Of note, to have minor changes on DNA isolation efficiency using various protocols, all of used protocols were performed tested in the similar situations and sites and by using the same tools and stock materials.

***DNA isolation protocols: ***Proposed method: The proposed method was based on protein digestion in two sequential steps. Every milliliter blood samples was dispensed in 3 ml of lysis buffer containing 6 M NaI (Sigma), 1% SDS, 10 mM EDTA, 20 mM Tris-HCL and proteinase K (20mg/ ml, sigma) and the mixture was incubated at 65 ˚C for 20 minutes in water bath. DNA was dehydrated in 70% ethanol and dissolved in ddH2O. Remained nucleoproteins were digested through incubating DNA molecules in 6 ml digestion buffer (50 mM Tris PH, 8.0, 10 mM EDTA, 0.05% SDS) including 20 mg/ml proteinase K for 20 minutes. DNA, was eventually eluted in ddH2O following two rounds of washing with 250 µl 70% ethanol.

DNA was also purified using QIAamp DNA Blood Mini Kit and AccuPrepTM Genomic DNA Extraction Kit according to their manufacturer’s instructions and then was dissolved in ddH2O. Due to required less starting material (< 1ml) for both kit based protocols, we had to isolate DNA from one sample in multiple reactions according to instructions of each kit.

The quality of DNAs purified from above protocols was confirmed through amplification of *TSGA10* gene (9) by conventional PCR. To find the DNA extraction method with minimum small DNA fragments loss, DNA obtained product from each protocol was amplified with four genomic primers with small product sizes (< 500 bps) (Table 1). 

**Table 1:** Primer sequences used for confirmation of amplification of small DNA fragments

**Gene**	**Primer sequence**	**Amplicon size (bps)**
*TSGA10*-SNP273	Forward: 5´ GCACAGCGAGAAGAAATGAG 3´ Reverse: 5´CATTGCCAAACTCTCTCCAAGG 3´	129
*DFNB3-I *	Forward:5´ACAAACTTATATTCTTAGCACCTC3´ Reverse: 5´ ACACAAACAGAGCTGCTCATT 3´	250
*TSGA10*-SNP411	Forward: 5´CAGATGCTGATTGCAGTCTTTG 3´ Reverse: 5´AATCTGTTACCCTCTGCCTCAG 3´	337
*TSGA10*-SNP	Forward: 5´ ATTTGGTAAGAAGGAGGGACA 3´ Reverse: 5´CCACCTCTTAGGCAAATCACA 3´	495

The products of both aforementioned PCR reactions regarding each isolation protocol were then resolved on agarose gel electrophoresis (2%).

***MeDIP-Real time PCR: ***To clearly determine the efficiency of each performed protocols in isolation of free fetal DNA, they should be separated from maternal DNA and being amplified. Methylated DNA immunoprecipitation (MeDIP) technique was implicated to at first enrich total methylated maternal and fetal free DNAs. Methylated fetal DNA sequences were then amplified using three specific primer pairs in real time PCR reaction. MeDIP-Real time PCR protocol was performed on all normal samples isolated by each three mentioned protocols according to previously reported method of analysis (Table 2) (10). 

Standard curve was drawn for three fetal specific sequence reactions using a DNA sample isolated from a whole blood sample with known concentration and copy number. All the three real time PCR programs were optimized as 40 cycles of 10 second at 95ºC following annealing and extension at 60 ºC for 30 seconds. Every Real time PCR reaction was included 2x SYBR Premix Ex Taq master mix (TaKaRa Bio Inc., Japan) and fetal specific primer pairs adjusted with nuclease free water up to total volume of 10 µl. Amplification was performed in a Rotor Gene 6000 Real time machine (Corbett, CA). Melt curve analysis was performed following each run of amplification to confirm the specificity of the amplified products and absence of primer dimer. Melting curve analysis was included 57°C for 15 sec which was followed by temperature increase to 95°C for 15 sec at the rate of 1°C per sec with continuous reading of fluorescence.

***Statistical analysis: ***The mean of total DNA concentrations and optical density (OD) in absorbance of 260/280 was determined by NanoDrop ND-1000 spectrophotometer and compared with each other using SPSS software (version 16, SPSS Inc., Chicago, IL, USA). In all the statistical analysis, α was considered to be 0.05 and ρ-value less than 0.05 was significant.

## Results

Due to the normalized pattern of all data using histogram analysis (p-value > 0.05), parametric tests were used to compare mean of parameters of three protocols with each other.

***DNA yield and quality:*** The mean of total DNA concentration, yield and purity (OD ratio of 260/280 nm/nm and OD ratio of 260/230 nm/nm are shown in Table 3. 

**Figure 1:**
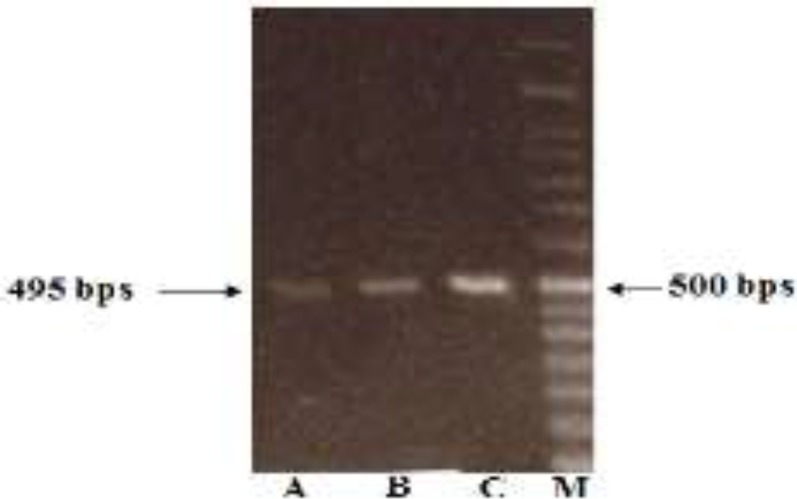
PCR amplification of DNAs obtained from each protocol; A: Qiagen Kit; B: Bioneer Kit; C: proposed method; M: DNA Marker (50 bps).

The mean of DNA yield obtained by our proposed was significantly higher than two kit based protocols (p<0.001). In using QIAamp DNA Blood Mini Kit according to manufacturer’s instruction without any modification, 15 samples failed to be amplified in the PCR reaction (Figure 1). 

**Table 2:** Primer pairs used to amplify fetal specific methylated sequences

**Fetal genes**	**Forward primer**	**Reverse primer**	**Amplicon size (bps)**
Sequence 1	5’-ATTCTCCACAGGGCAATGAG-3’	5’-TTATGTGGCCTTTCCTCCTG-3’	128
Sequence 2	5’-TGCAGGATATTTGGCAAGGT-3’	5’-CTGTGCCGGTAGAAATGGTT-3’	127
Sequence 3	5’-CCGTTATATGGATGCCTTGG-3’	5’-AAACTGTTGGGCTGAACTGC-3’	127

**Table 3:** NanoDrop characteristics of total DNAs obtained by five extraction protocols

**Isolation method**	**DNA concentration** **(ng/µl)**	**OD 260 /280** **(nm/nm)**	**OD 260/230** **(nm/nm)**
Suggested protocol	248.79 ± 14.07	1.62 ± 0.04	1.11 ± 0.14
AccuPrepTM Genomic DNA Extraction Kit	66.15 ± 15.42	1.69 ± 0.09	1.15 ± 0.13
QIAamp DNA Blood Mini Kit	46.26 ± 15.81	1.00 ± 0.07	0.6 ± 0.07

The best DNA purity was achieved through Bioneer (AccuPrepTM Genomic DNA Extraction) Kit (p-value < 0.001) (Table 3).

Minimum quantity and quality of DNA with significant deviation from other protocols was belonged to the Qiagen kit (p-value < 0.001).

**Figure 2:**
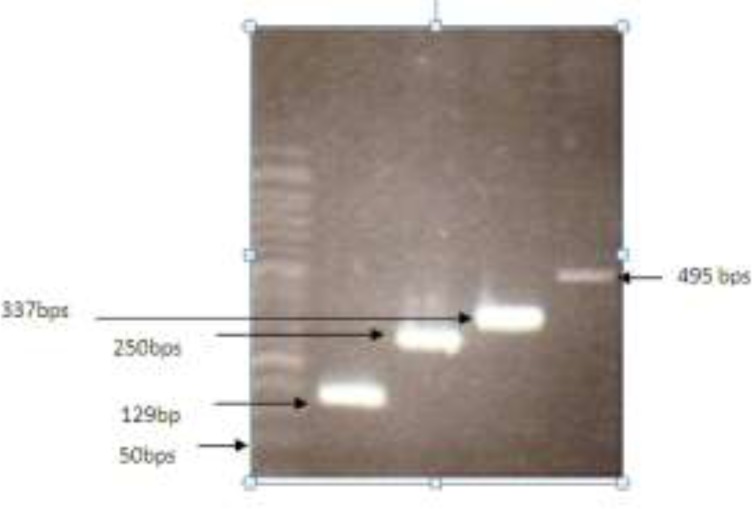
PCR amplification of DNAs obtained with our proposed method using various small genomic primers

PCR amplification: The efficiency of the proposed method of DNA extraction on isolation of small DNA fragments was revealed using four primer pairs designed to amplify different small DNA templates (Figure 2). DNAs from the four remaining methods were not amplified by genomic primers less than 400 bps in all of the samples.

***MeDIP Real time PCR: ***The mean of fetal specific DNA sequences concentrations was presented (Table 4) in which enrichment of cffDNA for all of three sequences in using of proposed protocol was significantly higher than two commercial kits (p = 0.01). The difference between Qiagen and Bioneer kits was meaningful for sequence 3 (p = 0.01) and in borderline significant difference for sequence 1 (p = 0.04).

## Discussion

cffDNA is usually fragmented and smaller relative to maternal free DNA, making them more amenable to be lost during the isolation process. Herein, it was demonstrated that the proposed method not only was superior to others in obtaining maximum total DNA yield, but also it was successful in enrichment of small DNA fragments and fetal specific DNA sequences. The inhibitory effect of NaI on nuclease enzymes and DNA oxidation and digestion of DNA associated proteins in two sequential steps may be the main mysteries behind keeping small fetal DNA fragments against loss during isolation process by suggested protocol. Following suggested protocol, the quantity of DNA obtained by Bioneer Kit was higher than Qiagen kit in both total and cffDNA isolation sections. Phenol/chloroform free characteristic of proposed protocol is another notable advantage due to its attenuating effects on Taq polymerase function and PCR efficiency as well as Real time PCR in further steps of analysis thanks to the presence of DNA adducts usually left with isolated DNA (11- 14). It seems that the proposed protocol is quicker, cheaper and safer and had more DNA purity and yield owing to effective dissolving histon proteins in two digestion steps. Except of proteinase K which is the inevitable substance of every isolation protocol, it doesn’t require any expensive and hazardous materials as well as phenol and equipment. Moreover, all the process of isolation takes less than one hour accelerating the general diagnostic procedure.

**Table 4:** Average concentration of three fetal sequences isolated by each protocol

**Isolation method**	**Sequence 1** **(ng/ml)**	**Sequence 2** **(ng/ml)**	**Sequence 3** **(ng/ml)**
Proposed protocol	7.4 ± 1.14	6.9 ± 1.04	8.5 ± 1.14
AccuPrepTM Genomic DNA Extraction KitM	3.5 ± 0.72	2.69 ± 0.59	4.15 ± 0.83
QIAamp DNA Blood Mini Kit	1.9 ± 0.21	1.8 ± 0.17	2.05 ± 0.12

Qiagen kit based protocol had the least efficiency in recovery of cffDNA from maternal plasma amongst all the methods applied. Although, QIAamp DNA Blood Mini Kit was used for isolation of free DNA in some of the studies, some other reports have described its low efficiency compared with other isolation methods (15, 16). Fleischhacker et al. in two separate assays have demonstrated that QIAamp DNA Blood Mini Kit had the least DNA quantity compared with two other protocols, including Macherey & Nagel (NucleoSpin PlasmaF Kit), MagnaPure LCDNA isolation Kit and Nucleospin columns which is consistent with our results (17, 18). Moreover, Keshavarz et al. compared the efficiency of the THP protocol with QIAamp DNA Blood mini Kit in isolation of cffDNA from 25 and 10 plasma samples belonged to pregnant and non-pregnant women, respectively. They have found that DNA quantity isolated by THP manual method was significantly higher than QIAamp DNA Blood mini Kit (19). The quantity of cell free DNA using QIAamp DNA Blood mini Kit was demonstrated to be 2.7 fold lower than direct amplification of cfDNA without buffer treatment of samples (20). In contrast, in the previous study by Jorgez CJ et al. Qiagen Mini Kit has shown relatively high efficiency in isolation of cffDNA from maternal blood following magnetic-beads Kit (8). The Qiagen technical services have declared that their silica spin columns are unable to capture small DNA fragments smaller than 150 bps confining their strength in isolation of cffDNA (21). In the present study, inability to amplify small DNA fragments besides very lower concentration of fetal specific sequences was demonstrated for DNAs isolated from maternal blood using the Qiagen kit. 

Most of the studies focused on comparing free DNA isolation protocols were based on using various types of commercial Kits produced for isolation of total free DNA from plasma as well as viral DNA. 

In this study, the proposed protocol had significantly higher total and cell free fetal DNA concentration relative to Qiagen and Bioneer kit. This increment in DNA yield would have significant effect on the success rate of subsequent prenatal genetic analysis by enhancement of fetal DNA selection. Amplification of obtaining DNA in frame of templates smaller than 300 bps is strong evidence of recovery of small fetal DNA fragments usually lost with spin column based methods. Further modification may be warranted to increase the OD (260/280 and 260/230 nm/nm) of obtaining DNA to the optimal quality in favor of keeping DNA quantity to afford the best protocol for isolation of cffDNA. In addition, although, all the isolation processes for three used protocols were performed on the same place, the effect of isolation site would be tested in different laboratories with different situations and analytical tools. 

Taken together, our proposed method in the present study, could serve as a high efficiency and cost benefit alternative choice of cffDNA extraction in NIPD tests in all of populations with every financial supports. In addition, the proposed method could be an appropriate option to current methodologies and kit protocols used for isolation of free circulating DNA of cancer patients or viral diseases.

## Conclusion

Inhibitory effect of NaI on nucleases and double digestion of DNA associated proteins may be the main reasons behind the superiority of suggested protocol. Significantly higher amplification of fetal specific sequences in suggested protocol would be strong evidence on recovery of small fetal fragments as demonstrated with its superior total DNA quantity and its amplification in different PCR reactions.
